# Investigation of Behavioral Dysfunctions Induced by Monoamine Depletions in a Mouse Model of Parkinson's Disease

**DOI:** 10.3389/fncel.2018.00241

**Published:** 2018-08-08

**Authors:** Yong Li, Qian Jiao, Xixun Du, Mingxia Bi, Shuaishuai Han, Lingling Jiao, Hong Jiang

**Affiliations:** Department of Physiology, Shandong Provincial Key Laboratory of Pathogenesis and Prevention of Neurological Disorders and State Key Disciplines, Physiology, Qingdao University Medical College, Qingdao University, Qingdao, China

**Keywords:** Parkinson's disease, dopamine, noradrenaline, serotonin, motor symptoms, nonmotor symptoms

## Abstract

Parkinson's disease (PD) is characterized not only by typical motor symptoms, but also by nonmotor symptoms in the early stages. In addition to the loss of dopaminergic (DAergic) neurons, progressive degenerations of noradrenergic (NA) and serotonergic (5-HT) neurons were also observed. However, the respective effects and interactions of these monoamine depletions on certain nonmotor symptoms are still largely unknown. In the present study, we performed selective depletions of NA, 5-HT and DA in mice by intraperitioneal injection of N-(2-chloroethyl)-N-ethyl-2-bromobenzylamine hydrochloride (DSP-4), 4-chloro-L-phenylalanine (pCPA) and 1-methyl-4-phenyl-1,2,3,6-tetrahydropyridine (MPTP), respectively. DSP-4 led to a 34% decrease in the number of NAergic neurons in the locus coeruleus, and MPTP led to a 30% decrease in the number of DAergic neurons in the substantia nigra. Although there was no obvious change in the number of 5-HTergic neurons in the dorsal raphe nucleus after pCPA treatment, the levels of 5-HT and its metabolite in the frontal cortex and hippocampus were reduced, respectively. Locomotor activity deficit was induced by DA depletion and a decrease in traveled distance was potentiated by additional NA depletion. Despair-associated depressive-like behavior could be observed in every group. Anxiety states emerged only from the combined depletion of two or three monoamines. However, combined depletion of the three monoamines dramatically induced anhedonia, and it could also aggravate the depressive-like and anxiety behavior. Furthermore, NA depletion significantly reduced spatial learning and memory ability, which was not enhanced by additional 5-HT or DA depletion. Our data highlighted the interactive role of NA, 5-HT and DA in the motor, emotional and cognitive deficits, providing new insight into the complex orchestration of impaired monoaminergic systems that related to the pathology of PD.

## Introduction

Parkinson's disease (PD) is a common neurodegenerative disorder characterized by the loss of dopaminergic neurons in the substantia nigra (SN) and depletion of dopamine (DA) in the striatum. However, the depletion of DA *per se* is unable to cause the manifestation of both motor and nonmotor features of PD observed in different animal models (Wolters, 2009; Palmeri et al., 2017). Recently, increasing evidence implicated that other monoaminergic systems such as noradrenergic (NA) and serotonergic (5-HT) are also affected in the process of PD (Fornai et al., 2007; Kish et al., 2008). The degeneration of these neurons has been shown to play essential roles in the emergence of various clinical symptoms (Delaville et al., 2012). However, a specific role for each monoamine and their interaction in PD are still largely undetermined.

The widely accepted Braak's staging of PD has proposed a six-stage scheme that a characteristic Lewy pathology initially occurs in the lower brainstem and spreads an ascending course to the higher cortical structures (Braak et al., 2003). The following studies have reported that the pathology in regions adjacent to the medulla oblongata including the locus ceruleus (LC), a principal site of NA neurons, and the dorsal raphe nuclei (DRN), a main site of 5-HT neurons, occur at stage 1 or 2, whereas the SN neuronal loss occurs at stage 3 or 4 and induces a striking motor symptom (Braak et al., 2004; Burke et al., 2008). Nevertheless, the neuronal loss of NA has shown to be a greater extent than DA loss in PD patients (Buchman et al., 2012). A previous study showed that there were 83% loss of NA neurons and 78% loss of DA neurons in the PD brains (Zarow et al., 2003). Although the loss extent of 5-HT neurons in the DRN is under debate, 5-HT concentrations in the putamen and caudate nucleus of dorsal striatum are highly reduced by 51–66% in PD patients (Kish, 2003; Kish et al., 2008).

Several studies have reported a correlation between the severity of NA, 5-HT, and DA depletions and worsening of PD neurological symptoms (Delaville et al., 2012; Faggiani et al., 2015). Because of the widespread projections, LC NAergic system dominates a variety of neural circuits through releasing NA into the distinct brain regions, such as olfactory bulb, hippocampus, subthalamic nucleus, striatum and cerebral cortex (Benarroch, 2009). The loss of NA may account for many nonmotor symptoms experienced by PD patients and animal models, including cognitive impairment (Del Tredici and Braak, 2013), sleep disorder (Kalaitzakis et al., 2013), depression and anxiety (Remy et al., 2005), and sympathetic autonomic failure (Palma and Kaufmann, 2014). Moreover, NA lesions in the LC also affects firing activity of the SN, which decreases DA release into the striatum, thus leading to the typical motor deficits associated with PD symptoms (Srinivasan and Schmidt, 2003; Masilamoni et al., 2017). In addition, a range of studies have reported that 5-HT plays a vital role in the development of depression and anxiety, which are recognized as other landmarks of the disease. For example, depression appears in about 45% of PD patients and considerably reduces the patient's quality of life (Reijnders et al., 2008). 5-HTergic innervations originate from the medial and dorsal RN, and that mainly projects to the basal ganglia, such as striatum. The neurodegeneration of 5-HT neurons leads to a defect of limbic function, a detrimental process which is not only involved in the motor control but also in the regulation of emotion and cognition (Teissier et al., 2017).

Since NA, 5-HT and DA have a prominent role in both pathophysiology process and phenotypical aspect of PD, we, therefore, investigated the respective effects and interactions of these monoamine depletions on motor and nonmotor symptoms including depressive-like, anxiety and cognitive dysfunctions in mice with serial neurotoxic monoamine lesions.

## Materials and methods

### Animals

Male C57BL/6 mice (8 weeks of age) were purchased from the Cavens Laboratory Animal Center (No. 201608441, Changzhou, China). All animals were maintained on a 12:12 h light/dark cycle under constant temperature (22°C) with food and water available *ad libitum*. The mice were allowed for acclimation in a colony room for 2 weeks and handled daily before starting experiments. This study was carried out in accordance with the recommendations of the National Institutes of Health Guide for the Care and Use of Laboratory Animals, and the protocol was approved by the Animal Ethical Committee of Qingdao University. Total 192 mice were randomly divided into eight groups, comprising a saline-treated group, and NA, 5-HT, DA, NA/5-HT, NA/DA, 5-HT/DA as well as NA/5-HT/DA depleted group (*n* = 24 in each group). To avoid interference between multiple test performances, each group was divided into three subgroups, which contained 8 mice. Animals in the first subgroup were used for open field test, sucrose preference test and forced swim test; mice in the second subgroup were underwent bar test, elevated plus maze and tail suspension tests; mice in the third subgroup were used for the Morris water maze assay. Afterwards, 12 mice from each group were randomly selected for tissue content determination, and another 12 mice for immunohistochemical analysis.

### Drug administration for monoamine depletions

**Figure 1:**
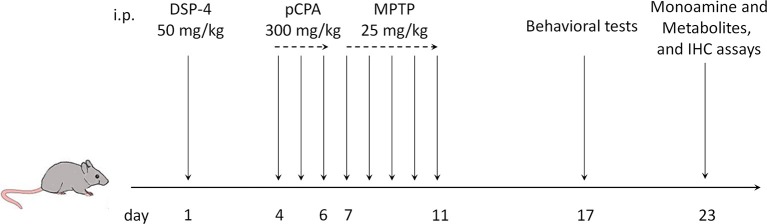
Time course of drug injection, behavior tests and neurochemical analyses. i.p., intraperitoneal injection; DSP-4, N-(2-chloroethyl)-N-ethyl-2-bromobenzylamine hydrochloride; MPTP, 1-Methyl-4-phenyl-1,2,3,6-tetrahydropyridine; pCPA, 4-chloro-L-phenylalanine.

NA depletion was obtained using N-(2-chloroethyl)-N-ethyl-2-bromobenzylamine (DSP-4, C8417, Sigma), a selective neurotoxin for NAergic projections originating from the LC. DSP-4 was dissolved in saline and intraperitoneally (i.p.) injected at a dose of 50 mg/kg according to the work (Grzanna et al., 1989). 5-HT depletion was achieved using 4-Chloro-D-phenylalanine (pCPA, C9419, Sigma), a selective inhibitor of 5-HT synthesis. pCPA was dissolved in saline and i.p. injected at a dose of 300 mg/kg during three consecutive days. This procedure was performed 3 days after DSP-4 treatment based on the occurrence of pathological deficits of NAergic system (Heal et al., 1993). 1-Methyl-4-phenyl-1,2,3,6-tetrahydropyridine (MPTP, M0896, Sigma), a neurotoxin to dopaminergic neurons in the SN, was dissolved in saline and injected in a subacute regimen (i.p., 25 mg/kg/day for 5 days) following the last pCPA injection. The vehicle-treated mice were administrated using saline in a comparable volume with the same protocol. The depletion of NA and/or 5-HT was executed prior to DA depletion according to the sequential Lewy pathology presented in PD patients (Braak et al., 2003). Behavioral tests were initiated one week after the last injection of MPTP or saline (Figure 1).

### Behavioral measurements

#### Open field test (OFT)

OFT was achieved between 9:00 a.m. and 1:00 p.m. in an isolated room, which was used to assess the spontaneous locomotor activity of animals. All mice were habituated for 30 min before the test. Individual mouse was placed in a square arena (27.3 × 27.3 × 20.3 cm) equipped with a camera. The test was lasted for 10 min. Total traveled distance and rearing were analyzed using an EthoVision XT video-tracking software (Noldus, Netherlands).

#### Sucrose preference test (SPT)

Sucrose consumption was conducted to evaluate anhedonia in rodents. Each mouse was individually housed in a separate cage and allowed free access to two bottles of water in the first day morning. In the second day morning, one bottle of water was changed to 1% sugar for another day. In the third day morning, the mice were deprived food and water until the lights turned off at 7:00 p.m., followed by supplying 1% sucrose and water in two bottles for 2 h. The position of the two bottles was always switched in the intermediate time. The weight of the bottles was measured before and after the test. Sucrose preference (%) = 100 × sucrose intake/(sucrose intake + water intake).

#### Bar test

The degree of catalepsy resulting from monoamine depletions was measured using the bar test by placing mice with both forepaws on a horizontal bar (diameter: 6 mm), which was set up 4.5 cm above the floor. The latency was recorded as the mouse grabbed the bar until their forepaws touched the floor or climbed over the bar. The cut-off of the test was set at 60 s.

#### Forced swim test (FST)

FST is the most commonly used method for monitoring despair-associated depression in rodents. Mice were individually forced to swim in a plastic cylindrical container (25 × 10 cm) irrigated with fresh water (25 ± 1°C) to a height of 15 cm. The duration of immobility was recorded over a period of 5 min. A mouse was judged as immobility when without struggling or only the rear feet slightly slide to keep its head above the water.

#### Tail suspension test (TST)

TST is another typical assay for evaluation of the despair-associated depressive behavior. Using adhesive tape to bind the tip of mice tail, each mouse was suspended 25 cm above the floor. The duration of immobility was measured manually during a total 5 min of test. A mouse was defined as immobility when minimal movements of the front legs instead of the hind legs. Furthermore, small oscillations and pendulum like swings which gained momentum from the earlier mobility were also consider as immobility (Zhang et al., 2017).

#### Elevated plus maze (EPM)

EPM was used to assay anxiety-related behavior. An applied apparatus is composed of two open arms and two closed arms (29 × 8 cm, with walls of 16 cm height), which was set up 30 cm above the floor. Each mouse was placed in the central square of the maze and allowed to explore the elevated plus maze for 5 min. The animals' movement track was recorded with EthoVision XT video-tracking software (Noldus, Netherlands). The percentage of time spent in open arms and numbers of arm entries were analyzed. Each arm was cleaned with alcohol before the next test.

#### Water maze training and spatial memory

Morris water maze test was performed to assess the spatial learning and memory skill of mice as described previously (Cui et al., 2016). The circular water maze pool (120 cm in diameter, 50 cm in depth) contained a movable platform (15 cm diameter), which was submerged 1 cm below the water surface. During training, each mouse will learn how to escape from the water by climbing the platform; if the mouse failed to find the platform within 60 s, it will be guided onto the platform by experimenter and allowed to stay for 30 s. Each mouse was subjected to four training trials each day. On day 5 and day 7 of training trials, the platform was removed and a probe test was performed to assay the spatial memory ability of mice. Animals' movement tracks in the training trials and the probe tests were recorded using an automated tracking system (Actimetrics, USA). The escape latency and the times crossing the platform were analyzed.

#### Biochemical determination of monoamine depletion

In order to validate the experimental model, tissular concentrations of the three monoamines as well as the metabolite 3,4-Dihydroxyphenylacetic acid (DOPAC) and homovanillic acid (HVA) of DA, the metabolite 3-Methoxy-4-hydroxyphenylglycol (MHPG) of NA, the metabolite 5-Hydroxyindoleacetic acid (5-HIAA) of 5-HT were determined by high performance liquid chromatography (HPLC) with electrochemical detection, as previously described (Shen et al., 2017). At the end of behavioral tests, 12 randomly selected mice per group were decapitated, and their brains were rapidly dissected. Both sides of the frontal cortex, hippocampus and striatum were weighed and homogenized in 300 μl liquid A (0.4 M HClO_4_) and centrifuged at 12,000 rpm for 30 min at 4°C. Eighty microliter of supernatant was mixed with 40 μl liquid B (20 mM potassium citrate, 300 mM K_2_HPO_4_, 2 mM disodium EDTA, and 2 octane sulfonic acid plus 7% methanol) and filtered through a 0.22 μm filter (Millipore, Germany). An electrochemical detector (Waters, USA) was operated in a screen mode. The flow rate was kept constant at 1 mL/min. The results were expressed as ng/g wet weight of the brain tissue.

#### Immunofluorescence labeling and stereological quantification

Another 12 mice per group were deeply anesthetized by 8% chloral hydrate, followed by ventricular perfusion with normal saline and 4% paraformaldehyde solution in 0.1 M PBS. Frozen brain blocks were coronally sectioned to 20 μm using a freezing microtome (Leica CM1900, Germany). After rinse with PBST (0.3% Triton-100 in 0.01 M PBS), sections were blocked by 10% goat serum for 30 min and then incubated with primary antibodies, dopamine beta-hydroxylase (DβH, ab209487, Abcam, 1:1,000), serotonin transporter (SERT, AB9726, Millipore, 1:1000) and tyrosine hydroxylase (TH, AB152, Millipore, 1:2000) for overnight at 4°C. Subsequently, the second antibody fluorophore-conjugated donkey anti-rabbit IgG H&L (Alexa flour 555, Invitrogen, 1:500) was added to incubate for 2 h at room temperature. After three times rinse, the sections were mounted with 70% glycerol. Stereological quantification was performed as previously described in our laboratory (Zhang et al., 2015). The number of DβH-, SERT- and TH-immunoreactive (ir) neurons was counted by optical fractionator unbiased stereological method using a fluorescent microscope (Zeiss, Germany) with Stereo Investigator (Micro Bright Field, USA).

#### Statistical analyses

All statistical analyses were performed using GraphPad Prism Software (version 5). Escape latencies in Morris water maze for the individual trial and biochemical data were analyzed by two-way analysis of variance (ANOVA) with repeated measures. Statistical analyses for the rest of the data were performed by one-way ANOVA with Bonferroni's multiple comparison tests. All data were shown as mean ± SEM. *p* < 0.05 is considered statistically significant.

## Results

### Monoamine contents and turnover are altered by selective drug administrations

The tissue contents of NA, 5-HT and their metabolites in the frontal cortex and hippocampus, as well as DA and its metabolites in the frontal cortex and striatum in eight groups were quantified by post-mortem HPLC.

DSP-4 is a selective neurotoxin that is widely used to lower brain NA to investigate the functions of the central noradrenergic system (Grzanna et al., 1989). In this study, DSP-4 injection dramatically decreased by 60% tissue level of NA [one-way ANOVA, *F*_(7, 72)_ = 28.61, *p* < 0.001, Table 1) and by 36% tissue level of MHPG [*F*_(7, 72)_ = 8.54, *p* < 0.05] in the frontal cortex, and decreased by 58% of NA [*F*_(7, 69)_ = 25.82, *p* < 0.001] and by 33% of MHPG [*F*_(7, 69)_ = 6.68, *p* < 0.05] in the hippocampus compared to saline-treated animals. Notably, significantly decreased NA and MHPG levels in the frontal cortex and hippocampus were also observed in DSP-4/pCPA, DSP-4/MPTP and DSP-4/pCPA/MPTP groups [two-way ANOVA, *F*_(3, 76)_ = 40.50 and *F*_(3, 72)_ = 35.49, *p* < 0.001, respectively]; however, these decreased values were similar to that in DSP-4 group, suggesting that pCPA and/or MPTP injections did not modify by themselves tissue content of NA and its metabolite in DSP-4-injected mice. A ratio of MHPG/NA represents the metabolic rate of NA. Furthermore, we found that DSP-4 treatment significantly increased MHPG/NA ratios in the frontal cortex and hippocampus [*F*_(7, 66)_ = 9.13 and *F*_(7, 70)_ = 7.43, *p* < 0.05, respectively], suggesting that DSP-4 has an effect on NA metabolism.

**Table 1:** Tissue contents of NA and MHPG (represents in ng/g of tissue) in the frontal cortex and hippocampus of mice brain were measured by HPLC, and the ratios of MHPG/NA were analyzed after DSP-4, pCPA, and MPTP administration respectively.

**Treatments**	**Frontal cortex**			**Hippocampus**		
	**NA**	**MHPG**	**MHPG/NA**	**NA**	**MHPG**	**MHPG/NA**
Saline (*n* = 12)	224.3 ± 16.1	57.3 ± 3.5	0.25 ± 0.03	303.7 ± 22.1	104.6 ± 12.6	0.34 ± 0.07
DSP-4 (*n* = 10)	88.5 ± 7.6^***^	36.8 ± 4.9^*^	0.43 ± 0.09^*^	125.4 ± 19.4^***^	69.5 ± 10.6^*^	0.55 ± 0.09^*^
pCPA (*n* = 12)	212.7 ± 18.4	46.9 ± 2.7	0.21 ± 0.04	284.9 ± 26.6	106.7 ± 14.2	0.37 ± 0.04
MPTP (*n* = 9)	228.5 ± 20.6	45.1 ± 4.5	0.19 ± 0.05	315.8 ± 20.9	89.2 ± 9.5	0.28 ± 0.04
DSP-4/pCPA (*n* = 11)	79.5 ± 9.4^***^	39.6 ± 3.8^*^	0.51 ± 0.11^*^	127.2 ± 15.5^***^	63.2 ± 5.3^*^	0.49 ± 0.07^*^
DSP-4/MPTP (*n* = 9)	91.7 ± 8.4^***^	45.4 ± 3.1	0.48 ± 0.05^*^	131.5 ± 16.9^***^	75.6 ± 8.5^*^	0.57 ± 0.08^*^
pCPA/MPTP (*n* = 10)	201.5 ± 23.4	53.6 ± 6.7	0.26 ± 0.07	296.4 ± 36.7	92.7 ± 7.3	0.31 ± 0.03
DSP-4/pCPA/MPTP (*n* = 11)	66.7 ± 10.4^***^	32.7 ± 5.1^**^	0.49 ± 0.06^*^	116.3 ± 20.1^***^	78.2 ± 7.9^*^	0.67 ± 0.12^**^

*All values are expressed as mean ± S.E.M*.

* *p < 0.05*,

** *p < 0.01*,

*** *p < 0.001 in comparison with the saline group*.

PCPA has been used extensively to target serotonergic afferents and reduce overall 5-HT in the rodent brain (Kornum et al., 2006). PCPA injection significantly decreased by 53% tissue level of 5-HT [one-way ANOVA, *F*_(7, 72)_ = 35.74, *p* < 0.001, Table 2] and by 73% tissue level of 5-HIAA [*F*_(7, 72)_ = 27.31, *p* < 0.001] in the frontal cortex, and decreased by 57% of 5-HT [*F*_(7, 69)_ = 38.32, *p* < 0.001] and by 76% of 5-HIAA [*F*_(7, 69)_ = 45.64, *p* < 0.001] in the hippocampus compared to saline-treated animals. Similarly, significantly decreased 5-HT and 5-HIAA levels in the frontal cortex and hippocampus were also observed in DSP-4/pCPA, pCPA/MPTP and DSP-4/pCPA/MPTP groups when compared to saline-treated group [two-way ANOVA, *F*_(3, 80)_ = 23.46 and *F*_(3, 78)_ = 36.14, *p* < 0.001, respectively]; however, these decreased values were similar to that in pCPA group, suggesting that DSP-4 and/or MPTP injections did not modify by themselves tissue content of 5-HT and its metabolite compared to pCPA-injected mice. A ratio of 5-HIAA/5-HT represents the metabolic rate of 5-HT. We found that the ratio of 5-HIAA/5-HT was significantly decreased in the frontal cortex and hippocampus after pCPA treatment [*F*_(7, 72)_ = 10.75, *p* < 0.05 and *F*_(7, 69)_ = 18.48, *p* < 0.01], suggesting that pCPA could decrease the metabolism of 5-HT.

**Table 2:** Tissue contents of 5-HT and 5-HIAA (represents in ng/g of tissue) in the frontal cortex and hippocampus of mice brain were measured by HPLC, and the ratios of 5-HIAA/5-HT were analyzed after DSP-4, pCPA, and MPTP administration respectively.

**Treatments**	**Frontal cortex**			**Hippocampus**		
	**5-HT**	**5-HIAA**	**5-HIAA/5-HT**	**5-HT**	**5-HIAA**	**5-HIAA/5-HT**
Saline (n = 12)	443.1 ± 19.7	275.6 ± 22.4	0.62 ± 0.11	379.2 ± 24.5	375.2 ± 25.9	0.99 ± 0.14
DSP-4 (*n* = 10)	447.3 ± 19.9	349.5 ± 21.3	0.78 ± 0.15	353.4 ± 22.7	427.1 ± 33.6	1.21 ± 0.21
pCPA (*n* = 12)	205.9 ± 33.7^***^	74.8 ± 9.2^***^	0.36 ± 0.05^*^	164.5 ± 24.9^***^	87.9 ± 5.9^***^	0.53 ± 0.08^**^
MPTP (*n* = 9)	435.1 ± 19.6	253.3 ± 17.3	0.58 ± 0.09	366.9 ± 28.8	325.7 ± 23.8	0.89 ± 0.11
DSP-4/pCPA (*n* = 11)	182.6 ± 37.5^***^	123.8 ± 10.5^***^	0.67 ± 0.09	166.6 ± 20.7^***^	153.1 ± 11.2^***^	0.91 ± 0.18
DSP-4/MPTP (*n* = 9)	419.3 ± 19.1	255.6 ± 14.2	0.61 ± 0.13	339.7 ± 28.4	285.7 ± 18.4	0.84 ± 0.07
pCPA/MPTP (*n* = 10)	191.8 ± 34.7^***^	79.3 ± 4.8^***^	0.41 ± 0.06^*^	171.8 ± 24.0^***^	81.3 ± 9.2^***^	0.47 ± 0.05^**^
DSP-4/pCPA/MPTP (*n* = 11)	178.5 ± 37.7^***^	69.4 ± 5.6^***^	0.39 ± 0.03^*^	163.2 ± 27.4^***^	74.3 ± 5.5^***^	0.45 ± 0.07^**^

*All values are expressed as mean ± S.E.M*.

**#x0002A;:** *p < 0.05*,

** *p < 0.01*,

*** *p < 0.001 in comparison with the saline group*.

The neurotoxin MPTP is the most commonly used to damage dopaminergic system in mice for PD model (Blume et al., 2009). The treatment of MPTP induced a significant decreased tissue levels of DA [78%, *p* < 0.001, one-way ANOVA, *F*_(7, 65)_ = 43.81, Table 3], DOPAC [44%, *F*_(7, 65)_ = 33.96, *p* < 0.001] and HVA [46%, *F*_(7, 65)_ = 39.42, *p* < 0.001] in the striatum but not in the frontal cortex. The additional treatment of DSP-4 and/or pCPA to MPTP further aggravated the depletion of DA and its metabolite contents in the striatum. It showed that the contents were decreased by 43% for DA, 46% for DOPAC and 57% for HVA in DSP-4/MPTP group, by 34% for DA, 38% for DOPAC and 57% for HVA in pCPA/MPTP group, and by 58% for DA, 52% for DOPAC as well as 64% for HVA in DSP-4/pCPA/MPTP group compared to MPTP group [*F*_(7, 67)_ = 31.64, *p* < 0.001], respectively. Consistently, a ratio of (DOPAC+HVA)/DA reflecting the metabolic rate of DA did not differ in the frontal cortex between MPTP group and saline group, while it was increased in DSP-4/pCPA/MPTP group [*F*_(7, 63)_ = 19.94, *p* < 0.01]. In striatum, (DOPAC+HVA)/DA ratio was significantly increased after MPTP treatment [*F*_(7, 68)_ = 17.62, *p* < 0.01], suggesting that MPTP could increase striatal DA metabolism.

**Table 3:** Tissue contents of DA, DOPAC, and HVA (represents in ng/g of tissue) in the frontal cortex and striatum of mice brain were measured by HPLC, and the ratios of (DOPAC + HVA)/DA were analyzed after DSP-4, pCPA, and MPTP administration respectively.

**Treatments**	**Frontal cortex**				**Striatum**			
	**DA**	**DOPAC**	**HVA**	**(DOPAC+HVA)/DA**	**DA**	**DOPAC**	**HVA**	**(DOPAC+HVA)/DA**
Saline (*n* = 12)	521.7 ± 45.8	32.8 ± 2.6	48.9 ± 3.1	0.15 ± 0.06	7,020 ± 551.4	617.7 ± 56.8	997.8 ± 78.2	0.23 ± 0.04
DSP-4 (*n* = 10)	506.4 ± 27.9	30.3 ± 4.3	43.1 ± 5.3	0.14 ± 0.05	6,742 ± 600.7	553.8 ± 58.4	889.9 ± 96.3	0.21 ± 0.08
pCPA (*n* = 12)	531.5 ± 28.4	34.4 ± 2.9	56.5 ± 6.1	0.17 ± 0.05	6,574 ± 391.6	698.2 ± 67.3	941.4 ± 88.5	0.25 ± 0.08
MPTP (*n* = 9)	496.9 ± 44.1	36.8 ± 5.1	69.5 ± 5.6	0.21 ± 0.07	1,494 ± 155.8^***^	347.9 ± 25.7^***^	543.5 ± 63.1^***^	0.59 ± 0.12^**^
DSP-4/pCPA (*n* = 11)	507.5 ± 44.6	28.9 ± 3.5	43.6 ± 4.4	0.14 ± 0.06	7,175 ± 532.4	603.7 ± 67.6	897.8 ± 74.4	0.21 ± 0.04
DSP-4/MPTP (*n* = 9)	473.9 ± 20.5	34.1 ± 3.6	52.1 ± 3.7	0.18 ± 0.06	847.7 ± 71.0^***^^##^	189.1 ± 23.1^***^	235.6 ± 21.3^***^	0.51 ± 0.11^**^
pCPA/MPTP (*n* = 10)	474.8 ± 28.9	31.7 ± 2.9	42.6 ± 3.2	0.15 ± 0.04	977.2 ± 105.6^***^^#^	213.3 ± 24.4^***^	231.2 ± 18.4^***^	0.45 ± 0.12^*^
DSP-4/pCPA/MPTP (*n* = 11)	127.1 ± 15.9^***^	10.7 ± 1.2^***^	24.6 ± 2.1^**^	0.28 ± 0.08^**^	620.4 ± 81.3^***^^###^	165.4 ± 19.9^***^	193.2 ± 15.7^***^	0.57 ± 0.14^**^

*All values are expressed as mean ± S.E.M*.

* *p < 0.05*,

** *p < 0.01*,

*** *p < 0.001 in comparison with saline group*;

# *p < 0.05*,

## *p < 0.01*,

### *p < 0.001 in comparison with MPTP group*.

### Effects of monoamine depletions on locomotor activity

**Figure 2:**
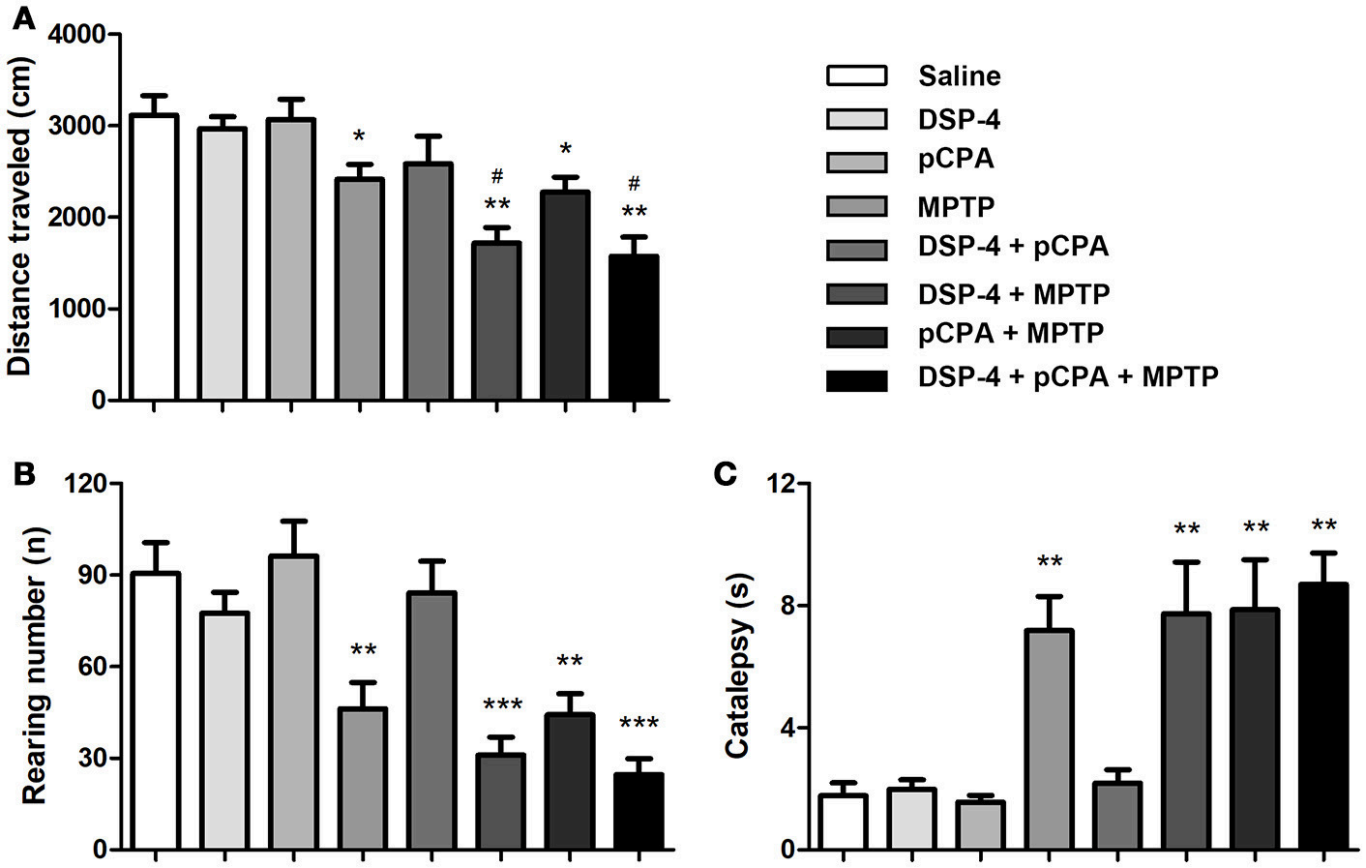
Monoaminergic depletion induced changes in locomotor activity and catalepsy. The total distance traveled **(A)** and number of rearing **(B)** during 10 min was reduced by MPTP treatment in the OFT. **(C)** MPTP-induced DA depletion promoted catalepsy in the Bar test. Each value represents mean ± SEM. *p < 0.05, **p < 0.01, ***p < 0.001 in comparison with the saline group, and ^#^*p* < 0.05 in comparison with the MPTP group was assessed using one-way ANOVA followed Bonferroni's tests. *N* = 8 in saline group, *n* = 6 in MPTP group, *n* = 7 in DSP-4 group, *n* = 8 in pCPA group, *n* = 7 in DSP-4/pCPA group, *n* = 6 in DSP-4/MPTP group, *n* = 8 in pCPA/MPTP group, *n* = 7 in DSP-4/pCPA/MPTP group.

Consistent with previousstudies (Blume et al., 2009; Li et al., 2016), MPTP-induced DA depletion significantly decreased a total distance of spontaneous movement by 22% in the OFT compared to saline-treated mice [one-way ANOVA, *F*_(7, 51)_ = 8.81, *p* < 0.05, Figure 2A]. Depletion of NA and/or 5-HT did not affect locomotor activity. However, additional depletion of NA but not 5-HT potentiated the decreased total distance induced by DA depletion (with 29% reduction compared to DA-depleted mice, *p* < 0.05). Additionally, MPTP-induced DA depletion also decreased the number of rearing by 49% [one-way ANOVA, *F*_(7, 51)_ = 19.23, *p* < 0.01, Figure 2B), and similar decreases in rearing number could be seen in DSP-4/MPTP, pCPA/MPTP and DSP-4/pCPA/MPTP groups.

In addition to the locomotor activity, we determined whether monoaminergic depletions could promote catalepsy using the bar test. DA depletion significantly increased catalepsy time compared to saline-treated animals [one-way ANOVA, *F*_(7, 56)_ = 14.33, *p* < 0.01, Figure 2C]. Depletion of NA and/or 5-HT did not either trigger catalepsy or potentiate the increased catalepsy induced by DA depletion.

### Effects of monoamine depletions on depressive-like disorder and anxiety behavior

**Figure 3:**
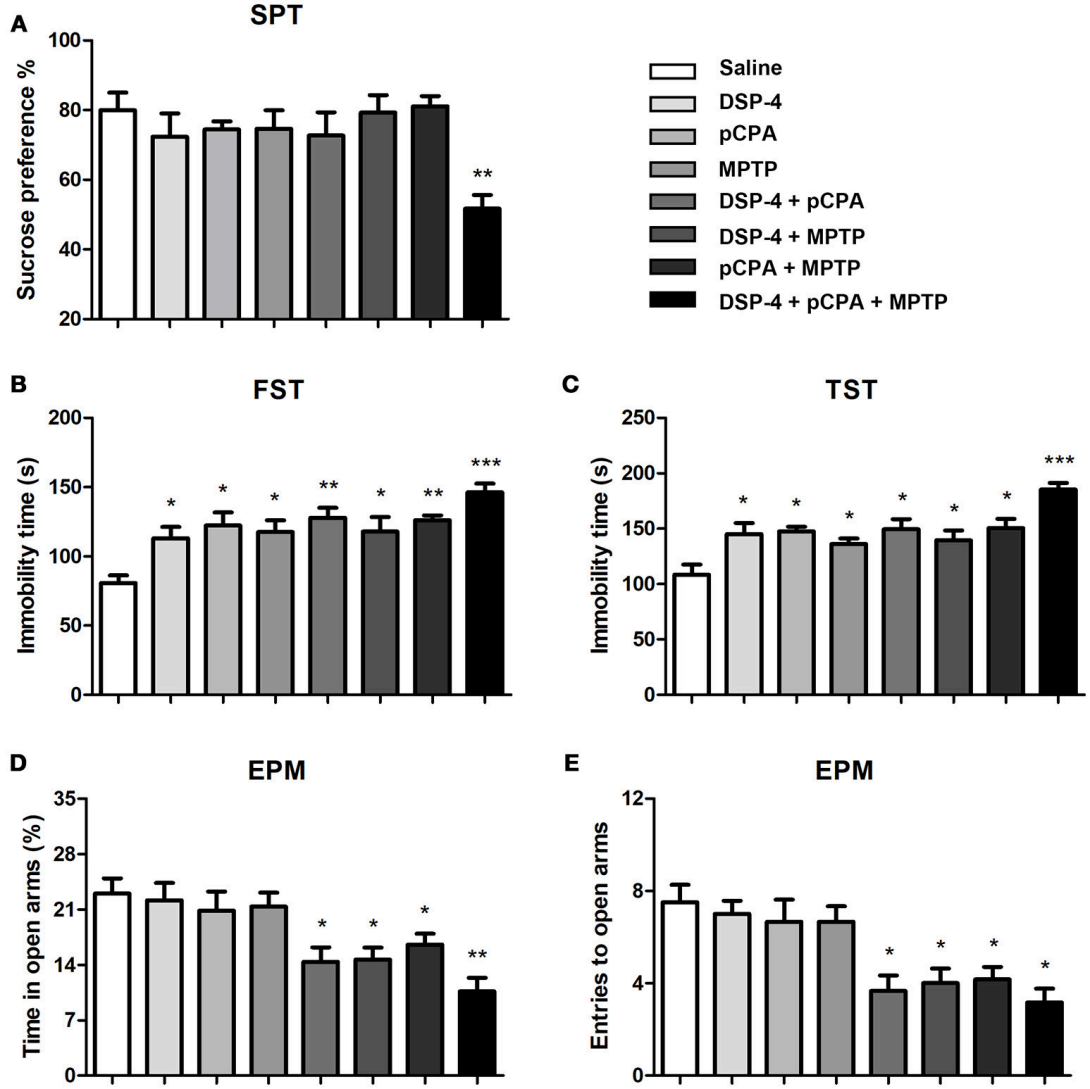
Monoaminergic depletions diversely affect anhedonia, depressive-like and anxiety behaviors. **(A)** Only combined depletions of all the three monoamines reduced the percentage of sucrose consumption measured by the SPT. **(B,C)** Despair-associated depression was measured by the FST and TST. All treated groups showed an increased duration of immobility during a 5 min measurement. **(D,E)** Anxiety was determined by the EPM. The time spent in open arms and number of open arms entries were decreased in group with combined any two or three neurotoxins treatment. Each value represents mean ± SEM. All data were analyzed using one-way ANOVA followed, when significant, by Bonferroni's tests. *p < 0.05, **p < 0.01, ***p < 0.001 in comparison with the saline group. The number of mice used in SPT and FST were same with that in OFT. For TST and EPM, *n* = 8 in saline group, *n* = 7 in DSP-4 group, *n* = 8 in pCPA group, *n* = 6 in MPTP group, *n* = 6 in DSP-4/pCPA group, *n* = 6 in DSP-4/MPTP group, *n* = 7 in pCPA/MPTP group, *n* = 7 in DSA-4/pCPA/MPTP group.

To evaluate mood behavioral disabilities we used different common tests. In SPT, mice with single depletion of NA or 5-HT or DA did not alter the sucrose consumption compared to saline-treated animals. However, only combined depletions of NA, 5-HT and DA induced an anhedonia behavior as the sucrose consumption was significantly decreased [one-way ANOVA, *F*_(7, 51)_ = 3.81, *p* < 0.01, Figure 3A].

In FST for despair-associated depression assay, our results showed that single depletion of NA, 5-HT, or DA significantly increased the immobility time compared to saline-treated animals. In addition, mice with depletion of NA or 5-HT combined with DA depletion also showed a significant increase in immobility time compared to saline-treated animals [one-way ANOVA followed by Bonferroni's multiple comparison test, *F*_(7, 53)_ = 5.5, *p* < 0.001, Figure 3B], although not significantly different from that in mice with depletion of monoamine alone. Interestingly, there is a trend toward an increased depressive-like behavior when combined depletions of the three monoamines. To further verify the effects of NA, 5-HT and DA on depression, we performed TST. A similar statistical result was obtained as for FST (Figure 3C).

Anxiety behavior was evaluated by EPM under NA, 5-HT or DA depletion. As shown, the time spent in open-arms and the number of entries were not affected by depletion of NA, 5-HT, or DA alone (Figures 3D,E), suggesting that NA, 5-HT and DA are insufficient by themselves to control anxiety behavior. However, depletions of any two of the monoamines induced a significant anxiety behavior (*p* < 0.05). Furthermore, combined depletions of the three monoamines seem to increase the anxiety behavior as the time spent in open-arms [one-way ANOVA, *F*_(7, 53)_ = 5.80, *p* < 0.001, Figure 3D] and the number of entries into open-arms [one-way ANOVA, *F*_(7, 53)_ = 6.55, *p* < 0.001, Figure 3E) were even lower.

### NA depletion-induced spatial learning and memory deficits

**Figure 4:**
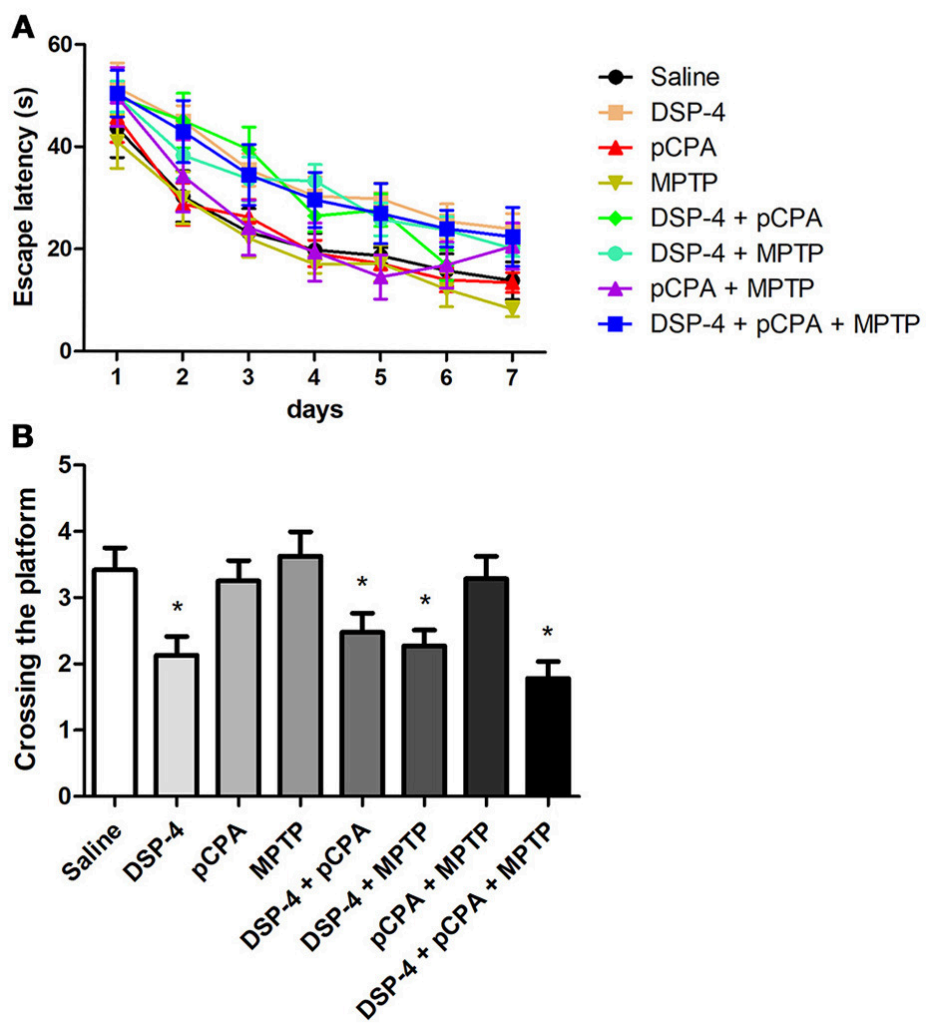
NA depletion reduced mice spatial learning and memory ability measured by the Morris water maze test. **(A)** Escape latency defined as time to find the hidden platform was increased in all DSP-4-treated groups compared to saline group on training day. **(B)** Platform crossing times during the probe test conducted 24 h after training were decreased not only in DSP-4 treatment group but also in groups of DSP-4 combined with pCPA and/or MPTP treatment. Each value represents mean ± SEM. *p < 0.05 in comparison with the saline group was analyzed using one-way ANOVA followed by Bonferroni's tests. *N* = 8 in saline group, *n* = 7 in DSP-4 group, *n* = 8 in pCPA group, *n* = 7 in MPTP group, *n* = 8 in DSP-4/pCPA group, *n* = 5 in DSP-4/MPTP group, *n* = 7 in DSP-4/pCPA group, *n* = 6 in DSP-4/pCPA/MPTP group.

To examine the changes of spatial learning and memory deficits, the monoamine-lesioned mice were trained and tested in Morris water maze. Our results showed that depletion of NA but not 5-HT or DA affects spatial learning during training as a tendency of the overall escape latency to hidden platform was significantly delayed in contrast to that of saline-treated group [two-way ANOVA, *F*_(1, 14)_ = 8.93, *p* < 0.001, Figure 4A]. Additional depletions of 5-HT and/or DA failed to induce any potentiation of the learning impairment caused by NA depletion. In probe test, the number of platform crossing was significantly decreased in NA-depleted group compared to the controls [one-way ANOVA, *F*_(3, 25)_ = 4.93, *p* < 0.05, Figure 4B], indicating NA-depleted mice had a spatial memory deficit. Similarly, combining 5-HT and/or DA depletions with NA depletion did not aggravate the decrease in the number of platform crossing.

### Toxic effect of DSP-4, pCPA, and MPTP on monoaminergic neurons in the mice brain

**Figure 5:**
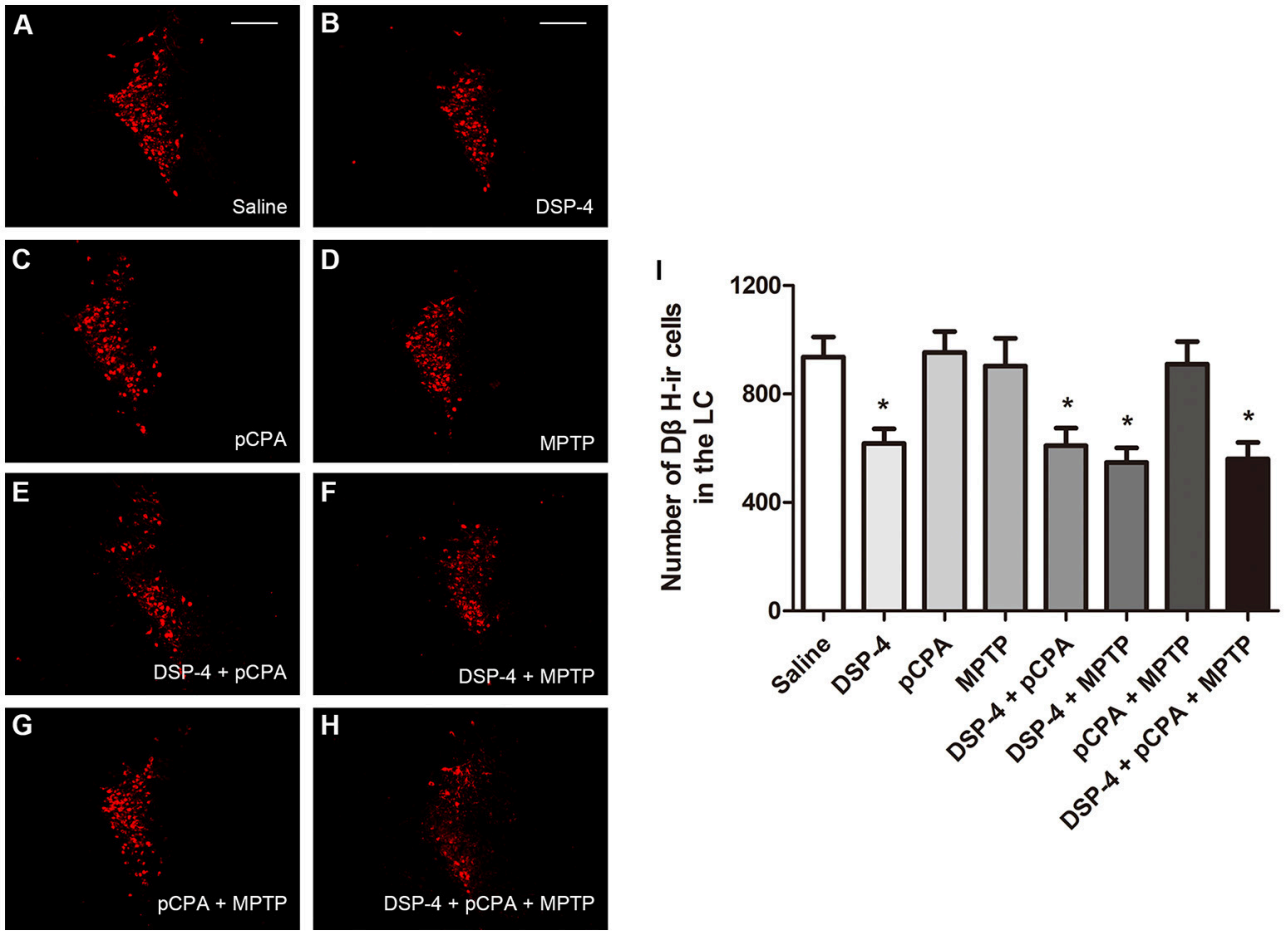
The number of DβH-immunostaining cells in the LC was reduced by neurotoxin treatments. **(A–H)** Micrographs of DβH-ir cells in different groups were shown. **(I)** Stereological counting of DβH-ir cells in all groups. Decreased number of DβH-ir cells was observed in DSP-4 group and DSP-4 along with pCPA and/or MPTP treated groups. Each value represents mean ± SEM. All data were analyzed using one-way ANOVA, *n* = 9. *p < 0.05 in comparison with the saline group. Scale bars = 100 μm.

**Figure 6:**
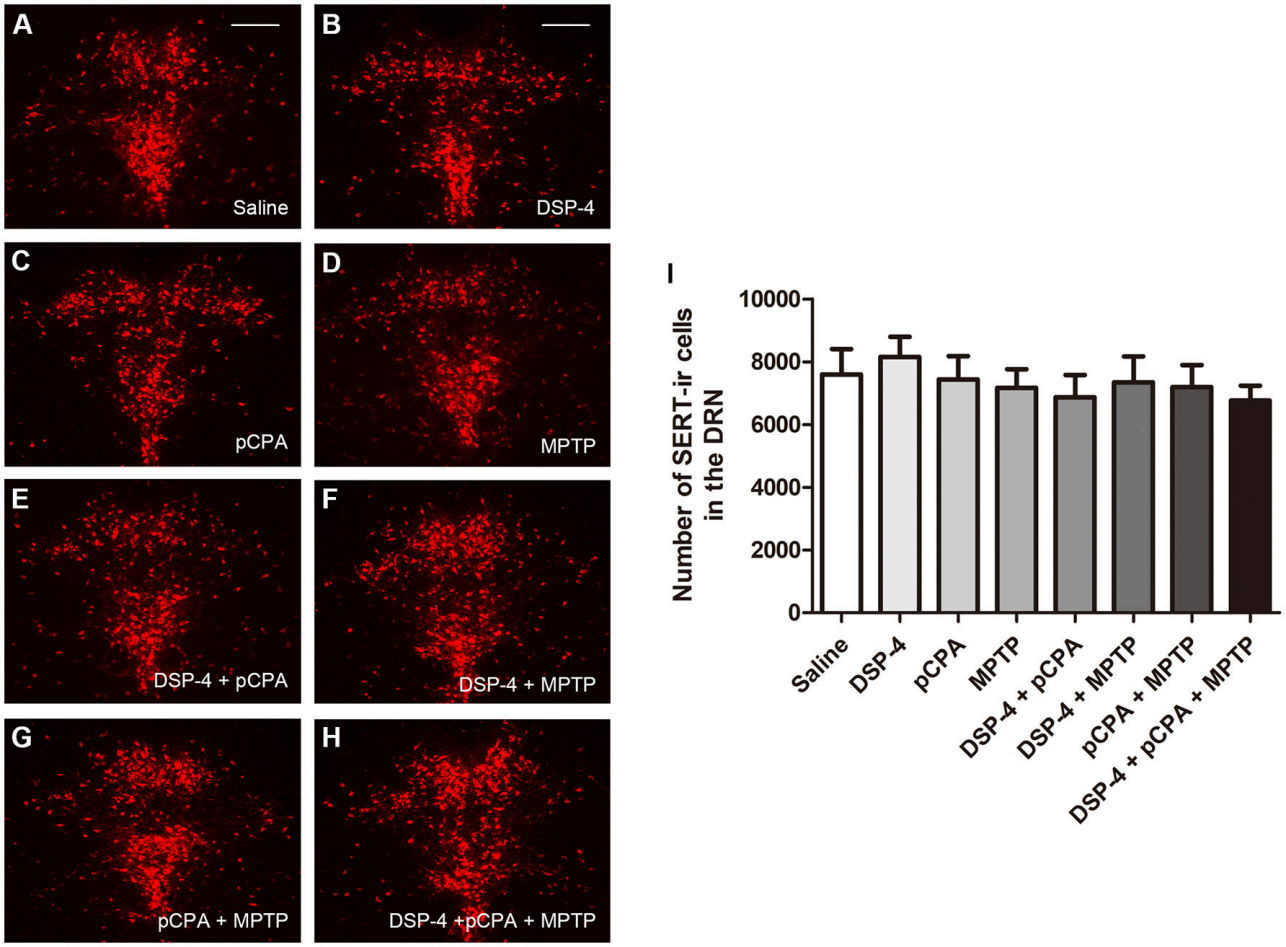
The number of SERT-immunostaining cells in the DRN was unchanged by neurotoxin treatments. **(A–H)** Micrographs of SERT-ir cells in different groups were shown. **(I)** Stereological counting of SERT-ir cells in all groups. There is no changed number of SERT-ir cells observed in all treatment groups. Each value represents mean ± SEM. All data were analyzed using one-way ANOVA, *n* = 9. Scale bars = 100 μm.

**Figure 7:**
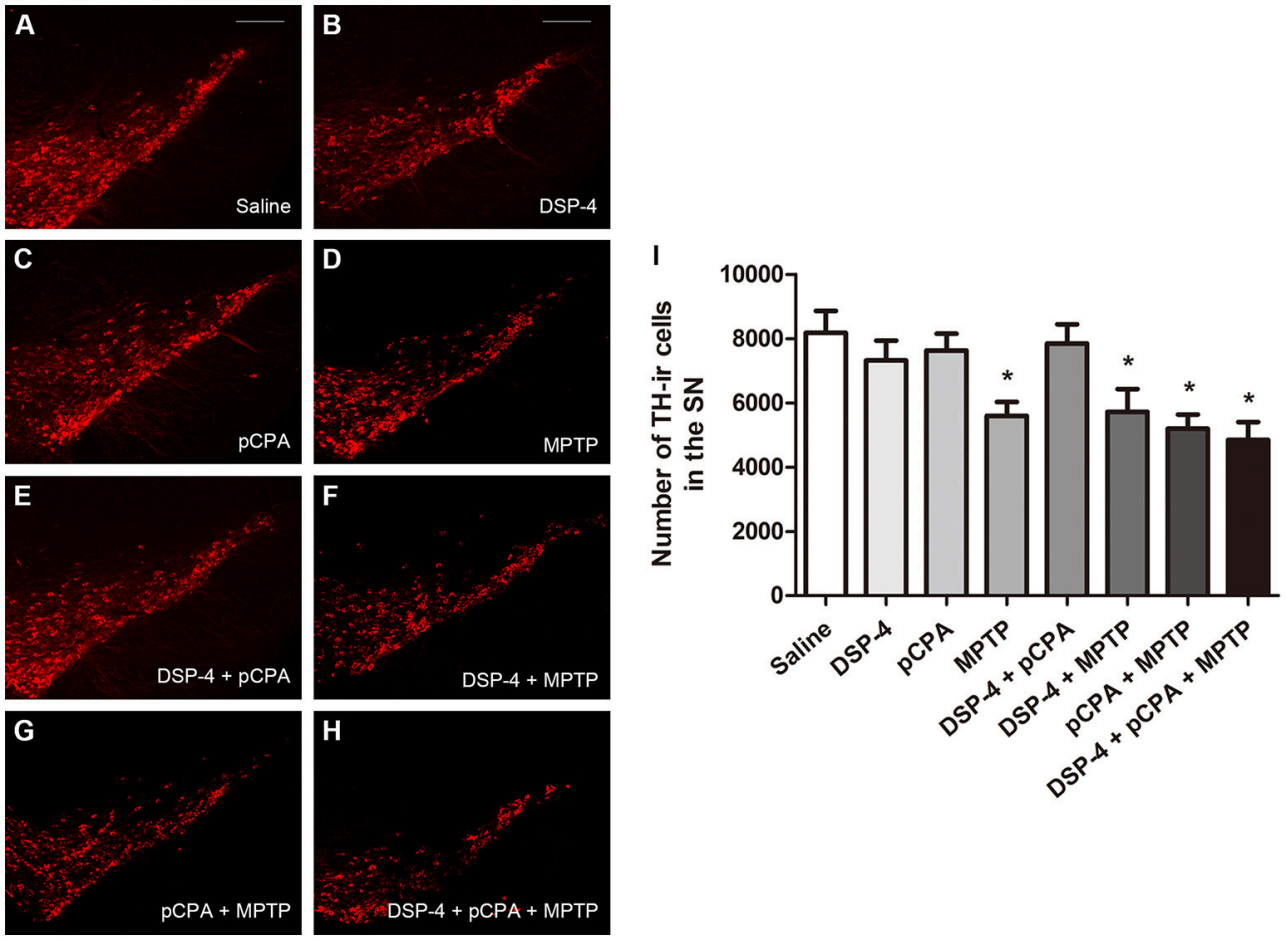
The number of TH-immunostaining cells in the SNpc was reduced by neurotoxin treatments. **(A–H)** Micrographs of TH-ir cells in different groups were shown. **(I)** Stereological counting of TH-ir cells in all groups. Decreased number of TH-ir cells was observed in MPTP group and MPTP together with DSP-4 and/or pCPA groups. Each value represents mean ± SEM. All data were analyzed using one-way ANOVA, *n* = 9. *p < 0.05 in comparison with the saline group. Scale bars = 100 μm.

Using immunofluoresent staining, we evaluate whether the toxic effects of the three neurotoxins are associated with the survival of monoaminergic neurons. DβH catalyzes the conversion of DA to NA, and it is expressed and identified as a marker in NAergic neurons. As shown, the number of DβH-ir cells in the LC was reduced by 34% in DSP-4 group compared with saline group [one-way ANOVA, *F*_(7, 37)_ = 6.25, *p* < 0.001, Figure 5]. However, the percentage decrease in DβH-ir cells was no longer less in the groups of DSP-4 combined with other toxins. PCPA and MPTP injection alone or combined together had no effect on the survival of NAergic neurons in the LC (*p* > 0.05). SERT is a type of monoamine transporter protein that transports 5-HT from the synaptic cleft to the presynaptic neuron. The number of SERT-ir cells in the DRN was not changed in any treated groups compared with the saline-treated group [one-way ANOVA, *F*_(7, 37)_ = 0.36, *p* > 0.05, Figure 6]. TH catalyzes the rate limiting step in the synthesis of DA, and it is used as an important indicator of DAergic neurons in the SN. MPTP injection significantly reduced 30% TH-ir cells in the SN [one-way ANOVA, *F*_(7, 37)_ = 5.15, *p* < 0.001, Figure 7]. Similarly, the loss of TH-ir cells in MPTP group was parallel in the groups of MPTP accompanied DSP-4 and/or 5-HT injection.

## Discussion

There are a number of researches to focus on the PD models produced by DA depletion, because the classic pathology of PD is the progressive loss of DAergic neurons in the SNpc. However, new evidence has recently shown that NAergic and 5-HTergic transmitter systems are also extensively compromised in PD patients (Espay et al., 2014; Deusser et al., 2015). In contrast, these systems have received relatively few attentions in PD field, although their impairment could contribute to the early nonmotor symptoms including emotional and cognitive disorders (Wolters, 2009). In this study, we used three selective toxins to generate a novel mice model that mimic several behavioral and neurochemical depletions which present in the progression of a majority of PD patients (Braak et al., 2003).

For the motor performance, our results showed that DA depletion has been expected to induce a motor deficit, which is in line with many previous studies describing movement disorder after subacute MPTP injection (Blume et al., 2009; Li et al., 2016). In addition, 6-hydroxydopamine (6-OHDA)-lesioned rats were also demonstrated severe motor deficits. Nevertheless, our results showed 22% reduction in movement distance that was lower than 6-OHDA-induced 56% reduction of movements in rats (Delaville et al., 2012). The difference may be due to the depletion of DA in the striatum, which MPTP reduced by 78% DA in our study but 6-OHDA reduced by almost 95% tissue level of DA in the striatum (Delaville et al., 2012; Faggiani et al., 2015). Besides, it has been reported that NA depletion induced motor deficits that resemble to those observed after DA depletion (Rommelfanger et al., 2007). We demonstrated that mice with DSP-4 treatment alone failed to alter spontaneous movement, whereas NA depletion significantly aggravated the motor deficits induced by DA depletion in support of previous results obtained in mice model (Archer and Fredriksson, 2006) as well as rat model of PD (Srinivasan and Schmidt, 2003). In fact, DSP-4 treatment potentiated DA loss induced by MPTP, whereas this enhancement is only achieved when DSP-4-induced neurotoxic insult before MPTP treatment (Fornai et al., 1997; Thomas et al., 2007). The explanation might be that some neurotrophic factors, such as brain-derived neurotrophic factor and fibroblast growth factor, are down-regulated and thus result in a reduced protective effect of NA on DA neurons in the SN (Rommelfanger and Weinshenker, 2007). One previous study showed that stimulation of the NA neurons facilitates firing activity of DA neurons and striatal DA release (Belujon et al., 2007). Therefore, NA depletion decreased the activity of DA neurons and the transmission of nigro-striatal DA, which enhanced the consequences of DA depletion that observed in our study and others (Archer and Fredriksson, 2006; Belujon et al., 2007).

Depression is one of the most common psychiatric symptoms, and it has been demonstrated to occur prior to the onset of motor symptoms of PD (Ou et al., 2017). According to the natural phenomenon that mice prefer a sweetened solution rather than water, whereas depressed mice will no longer exhibit that preference, we performed SPT to assess an anhedonic state of animals (Branchi et al., 2008). We found that NA, 5-HT or DA depletion alone did not induce anhedonia but significant increased the despair-associated depression detected in the FST. Mice of distinct neurotoxin treated groups and controls showed an obvious preference for sucrose as they drank about 80% sucrose solution and 20% water, and such preference might cover the possible differences in anhedonia among the groups which may explain the discrepancy between the results measured in the SPT and FST. However, combined depletion of the three monoamines induced anhedonia and further lengthened the immobility time in the FST. In order to exclude the possibility that the depressive disposition of mice is owing to familiarity with the environment or an adaptive response to an inescapable situation (Nishimura et al., 1988), we reevaluated despair-associated depression of mice using TST and obtained consistent results with the FST. These data support the notion that depressive-like behavior reported in our study could result from the deficits of NA, 5-HT or DA as well as the interaction of the three monoaminergic systems.

Increasing evidence suggests dopaminergic deficits in the SNpc and ventral tegmental area contribute to the depressive behavior independently. DA depletion in nigrostriatal pathway influences limbic function through inducing a high-frequency stimulation of subthalamic nucleus (Gubellini et al., 2009). The similar abnormalities could be resulted from inhibiting the activity of 5-HTergic neuron (Temel et al., 2007). Moreover, 5-HT and its metabolites levels are decreased in several brain regions of PD patients with depression (Kish, 2003), suggesting the depletion of 5-HT contributes to the depressive behavior. Our results also showed that a decline of NA content is related with depressive behavior, which fit with the studies that a reduction in either LC pigmentation or NA transporter binding in the limbic regions leads to depression (Remy et al., 2005). In addition, the utilization of NA and/or 5-HT reuptake inhibitors as antidepressants further indicates the essential role of these monoamines in depressive behavior (Lee et al., 2010).

In contrast to the depressive-like behavior, depletion of NA, 5-HT, or DA alone did not lead to an anxiety behavior. However, the anxiety state of mice was induced by combined depletions of the two or three monoamines. Our data, to a certain extent, support the assumption that DA depletion alone was necessary but not sufficient to induce anxiety disorder (Gareri et al., 2002). However, these findings challenge the results that DA depletion alone decreased the number of entries into the open-arms (Tadaiesky et al., 2008). The discrepancy might be ascribed to the neurotoxin utilized in both studies. Tadaiesky et al. created rodent models using 6-OHDA injection, while we performed MPTP injection for DA depletion. It should be noticed that 6-OHDA as catecholaminergic neurotoxin lesioned not only DAergic neurons but also NAergic neurons (Glinka et al., 1997). There is one study reported that mice deficient in vesicular monoamine transporter 2 (VMAT2), a protein that transports neurotransmitters including NA, 5-HT and DA, exhibited obvious anxiety behavior (Taylor et al., 2009). Although the VMAT2-deficient mice have elucidated the association between the three monoamines and the anxiety behavior, it did not clarify which monoamine plays predominant role in the observed disorder.

Besides emotional deficits, we also observed a cognitive deficit in the form of impaired spatial learning and memory. This deficit is a result of NA depletion, since we did not find an impairment of spatial learning and memory in pure MPTP- or pCPA-treated groups. Our study and others showed the learning and memory impairment induced by NA depletion is similar to that achieved after bilateral inactivation of LC in both novel object recognition and Morris water maze (Khakpour-Taleghani et al., 2009). Loss of 5-HT innervations in the brain could lead to memory deficits. Tryptophan depletion has been shown to reduce 5-HT contents in the hippocampus and frontal cortex that impairs memory in novel object recognition test (Jenkins et al., 2010). However, it is noted that tryptophan depletion induced a chronic and more extensive lowering of 5-HT levels than our neurotoxin paradigm. These data suggest that NA and 5-HT play an important role in cognitive functions and their impairments have been noticing in PD. One of the major challenges for studying motor and nonmotor behaviors in PD models is that the nonmotor symptoms to some extent is affected by motor abilities. We present here that, for example, DSP-4-lesioned mice displayed obvious depression but without motor deficits; MPTP-lesioned mice induced dyskinesia, but they did not appear anxiety. Thus, our data at least partially indicate that some of the nonmotor behaviors delineated in monoamine-depleted mice are not affected by motor deficits.

DSP-4 treatment reduced the number of NAergic neurons in the LC and MPTP treatment reduced the number of DAergic neurons in the SN. Nevertheless, pCPA as a 5-HT synthesis inhbitor did not affect 5-HTergic neurons in the DRN, which may suggest that endogenous 5-HT synthesis is not a prerequisite for proliferation, differentiation and survival of 5-HTergic neurons (Gutknecht et al., 2012). It has long been known that MPTP can cause the degeneration of DAergic neurons in SN through inhibiting mitochondrial complex I of the electron transport chain. However, the toxic mechanism of DSP-4 to LC NAergic neurons is not fully clear. Previous studies reported that DSP-4 crosses the blood-brain barrier and is metabolized into a reactive aziridinium derivative, which is accumulated into the NAergic nerve terminals via NA transporter. The toxic aziridinium derivative reacts with unknown cellular components and thus destroys the terminals and finally leads to cell death (Ross and Stenfors, 2015). Nevertheless, we cannot overlook the possibility that DSP-4 may influence DβH expression in a few days after treatment (Fritschy and Grzanna, 1991). Regarding to DSP-4 potentiates the loss of DA levels in the striatum, we did not observe a further decrease in DAergic neurons in DSP-4/MPTP group compared to MPTP group, implicating that DSP-4 may affect the excitability of DAergic neurons and thus reduce the release of DA (Rommelfanger and Weinshenker, 2007).

## Conclusion

Our results provide new insights into the roles of NA, 5-HT, and DA in the performance of nonmotor and motor symptoms of PD. DA depletion in mice induced hypokinesia and additional depletion of NA enhanced the motor deficits. NA, 5-HT, or DA by themselves had an impact on depressive-like behavior. Anxiety behavior is induced by combined depletions of the two or three monoamines. Furthermore, a synergetic effect could be seen among the three monoamines on anhedonia, depression and anxiety. Depletion of NA rather than 5-HT or DA dramatically affected animals' spatial learning and memory ability. We, therefore, propose that in the context of experimental Parkinsonism the motor and nonmotor symptoms caused by monoamine lesions are more complicated than previously anticipant.

## Author contributions

YL and HJ conceived the project and designed the study. YL, SH, and LJ performed the experiments, analyzed data and interpreted results. YL wrote the manuscript. HJ, QJ, and XD reviewed and edited the manuscript. All authors have read and approved the final version of the manuscript.

### Conflict of interest statement

The authors declare that the research was conducted in the absence of any commercial or financial relationships that could be construed as a potential conflict of interest.

## Abbreviations

DSP-4: N-(2-chloroethyl)-N-ethyl-2-bromobenzylamine
pCPA: 4-Chloro-D-phenylalanine
MPTP: 1-Methyl-4-phenyl-1,2,3,6-tetrahydropyridine
NA: noradrenaline
MHPG: 3-Methoxy-4-hydroxyphenylglycol
5-HT: serotonin
5-HIAA: 5-Hydroxyindoleacetic acid
DA: dopamine
DOPAC: 3,4-Dihydroxyphenylacetic acid
HVA: homovanillic acid
LC: locus ceruleus
DRN: dorsal raphe nuclei
SN: substantia nigra
HPLC: high performance liquid chromatography
DβH: dopamine β-hydroxylase
SERT: serotonin transporter
TH: tyrosine hydroxylase
6-OHDA: 6-hydroxydopamine
PD: Parkinson's disease.
